# Assessing Hearing-Related Quality of Life in Adults With Hearing Loss: Validation of the German Cochlear Implant Quality of Life (CIQOL)-35 Profile

**DOI:** 10.1177/23312165261450792

**Published:** 2026-06-16

**Authors:** Elena Pützer, Theodore R. McRackan, Isabelle Boisvert, Judy R. Dubno, Karolin Schäfer

**Affiliations:** 1 Faculty of Humanities, Institute for Special Needs Education (d/Deaf and Hard of Hearing), 119884 University of Duisburg-Essen, Essen, Germany; 2 Faculty of Human Sciences, Department of Special Education and Rehabilitation, University of Cologne, Cologne, Germany; 3 Department of Otolaryngology—Head and Neck Surgery, 169841 Medical University of South Carolina, Charleston, SC, USA; 4 Faculty of Medicine and Health, Sydney School of Health Sciences, 522555 University of Sydney, Camperdown, NSW, Australia

**Keywords:** hearing loss, cochlear implant, quality of life, validation, patient-reported outcome measure

## Abstract

Hearing-related quality of life is a crucial outcome for adults with cochlear implants. The Cochlear Implant Quality of Life (CIQOL)-35 Profile is a patient-reported outcome measure originally developed in English. In a previous study, this instrument was cross-culturally adapted into German to address the need for standardized assessment tools in German-speaking regions. To ensure the adapted instrument meets psychometric criteria, validation is required. The German adaptation of the CIQOL-35 Profile was validated through an online survey including questions on demographics, the German CIQOL-35 Profile, and the German Nijmegen Cochlear Implant Questionnaire (NCIQ). The collected data were analyzed for reliability and validity. A total of 204 adults (aged 19–87 years) with bilateral hearing loss completed the online survey. Cronbach's α between 0.84 and 0.91 demonstrates good internal consistency for all subscales of the CIQOL-35 Profile and the global outcome. Moderate to strong correlations (*r_s_* = 0.55–0.85) between the CIQOL and NCIQ indicate good convergent validity. Confirmatory factor analysis established construct validity for the German CIQOL instruments. These findings confirm that the adapted German version of the CIQOL instruments is a reliable and valid measure for assessing hearing-related quality of life in adults with cochlear implants and demonstrates higher validity than the NCIQ. The availability of the CIQOL in multiple languages facilitates international comparability of research results and increases clinical application. Implementing quality of life instruments in clinical practice enables a more comprehensive evaluation of patient outcomes and may help identify patient needs that may be addressed in therapy and rehabilitation.

## Introduction

People with hearing loss do not only notice difficulties in hearing and communication but in multiple areas of life. Studies have shown that acquired hearing loss may have an impact on psycho-social aspects such as social activities and loneliness (Shukla et al., 2020). Associations between hearing loss and psychological comorbidities such as depressive symptoms and anxiety have also been reported (Blazer & Tucci, 2019; Loughrey et al., 2018). Moreover, there is some evidence for an association between hearing loss and cognitive decline—even dementia (Loughrey et al., 2018). The hearing loss itself and its side effects have an impact on the patients’ quality of life.

The concept of quality of life in relation to healthcare interventions has been increasingly emphasized over the last years. Based on the International Classification of Functioning, Disability, and Health (ICF) the focus has shifted from people's disabilities, to focusing on their level of health (World Health Organization [WHO], 2002). If the ICF is applied to the case of hearing loss, measurement of hearing loss using audiogram and speech recognition ability would only describe the body structure and function. However, disabilities that are experienced in activities or in real-world environments would be missing if only these measures are considered. In contrast, evaluation of quality of life provides a broader and more comprehensive assessment as it includes additional domains such as social engagement as well as personal and environmental factors.

There is increasing research activity that considers quality of life of people with hearing loss, for example for cochlear implant (CI) users. Recent findings suggest that health- and hearing-related quality of life improves for adult CI candidates, after cochlear implantation (Bekele Okuba et al., 2023; De Sousa et al., 2018; Gaylor et al., 2013; Häußler et al., 2019; Issing et al., 2020; McRackan et al., 2018; Philpott et al., 2025; Rostkowska et al., 2021; Sonnet et al., 2017). Researchers observe better quality of life after implantation for people who receive a CI for single-sided deafness as well (Lindquist et al., 2022; Muigg et al., 2020). Adults with bilateral hearing loss rate their quality of life higher if they are implanted with bilateral CIs than if implanted with unilateral CI (Crowson et al., 2017; Gaylor et al., 2013; Lewis et al., 2024; McRackan et al., 2018, 2019a). Significant improvements in quality of life after CI are also reported in older patients (>65 years) (Issing et al., 2020; Sonnet et al., 2017; Sorrentino et al., 2020). Adults with a prelingual onset of hearing loss perceive substantial quality of life benefits from CI, though speech recognition abilities may trail postlingually deafened adults (Canale et al., 2021; Straatman et al., 2014). However, variables that are known to contribute to the quality of life explain only a small percentage of the variance in quality of life scores and many factors that influence the quality of life after CI are still unknown (McRackan et al., 2019a). There are only weak correlations between speech recognition ability and quality of life (Capretta & Moberly, 2016; McRackan et al., 2018; Moberly et al., 2018; Philpott et al., 2025; Vasil et al., 2020), including speech recognition in noise (Berg et al., 2025; McRackan et al., 2018; Moberly et al., 2018), even if the changes between pre- and postoperative scores are considered in the analysis (McRackan et al., 2018; Philpott et al., 2025). This further emphasizes the need to measure quality of life when measuring CI user outcomes.

Pre- and postoperative measurements are used to evaluate and assure the effectiveness and quality of cochlear implantation. The individual's perception of their performance in daily life may differ from results from speech perception measures. Therefore, the assessment of patient-reported functional abilities should be included in evaluation of interventions with CI (American Academy of Audiology, 2019). As cochlear implantation does not only affect listening skills but also social and psychosocial areas, it is important to perform a broader outcome assessment, including quality of life measures (Capretta & Moberly, 2016; Rostkowska et al., 2021). Research into the effects of cochlear implantation on quality of life of patients and resulting insights will provide a means to optimize interventions for people with hearing loss. Therefore, it is necessary to assess quality of life during the intervention following the implantation.

Currently, to evaluate quality of life in CI users, researchers often use generic questionnaires such as the Health Utility Index (HUI-3), or questionnaires that were developed for adults who use hearing aids. Unfortunately, these instruments may not capture a comprehensive picture of all changes in quality of life that are specific to CI users. An advantage of generic questionnaires is that they can be used across patient populations, diseases, or interventions. However, disease-specific questionnaires are more sensitive to possibly important outcome changes than generic questionnaires (Andries et al., 2021). Questionnaires for people with hearing aids might miss certain aspects that are important for CI users because the magnitude of hearing loss and the hearing sensation and sound quality with CI are different from those with hearing aids (Andries et al., 2021; McRackan et al., 2017). Therefore, it is necessary to use quality of life instruments developed specifically for CI users to measure the social and psycho-social effects of cochlear implantation.

The Nijmegen Cochlear Implant Questionnaire (NCIQ) (Hinderink et al., 2000) is a commonly used questionnaire designed for the CI population. However, there is criticism of the topicality because items were developed more than 25 years ago and therefore some items are framed in a way that now appears obsolete (e.g., questions about contact to “deaf people”) and because of the methods that were used in the development and validation of the NCIQ (Andries et al., 2021; Lenarz et al., 2022). The length of 60 items was also criticized because it takes a long time to complete and score the questionnaire. Until recently, this was one of the few instruments to evaluate quality of life in the CI population (Andries et al., 2021), limiting researchers in choosing an adequate questionnaire for their studies.

In recent years, there have been some developments in the field of hearing-related quality of life instruments for adults. For example, a shortened version of the German NCIQ is now available (Weichbold et al., 2024). Some questionnaires address specific aspects of hearing-related quality of life such as the Music-Related Quality of Life Measure MuRQoL (Dritsakis et al., 2017) for the evaluation of music rehabilitation for adult CI users and the hearing-related quality of life questionnaire for auditory-visual, cognitive, and psychosocial functioning (hAVICOP) (Ceuleers et al., 2023). Three questionnaires were recently developed to evaluate overall hearing-related quality of life: (a) the Évaluation du Retentissement de la Surdité chez l’Adulte (ERSA) (Ambert-Dahan et al., 2018), (b) the Cochlear Implant Quality of Life (CIQOL) instruments (McRackan et al., 2019b), and (c) the Quality of Life in People with Hearing Loss Questionnaire (HL-QoL) (Illg et al., 2023). The ERSA includes 20 questions in four domains (quality of life, personal life, social life, and occupational life) that were developed by a multidisciplinary team. The HL-QoL consists of 21 questions and results in one overall quality of life score. Items were developed by an expert panel and linked to the biopsychosocial conceptual framework of the ICF.

### CIQOL Instrument Development

The CIQOL were specifically developed to capture quality of life changes in CI users by a team of researchers at the Medical University of South Carolina (McRackan et al., 2019b). The aim was to detect important topics to CI users and to phrase them into statements about different situations in everyday life that could be presented as items in a questionnaire (Table 1). A pool of items was developed with patient focus groups using grounded theory methods and based on recent standards for developing Patient-Reported Outcome Measures (PROMs) (McRackan et al., 2017). The initial item pool was refined through psychometric analyses, resulting in an item bank composed exclusively of items with sound psychometric properties (McRackan et al., 2019c). Two versions of the questionnaire were generated from the item bank using item response theory: the CIQOL-35 Profile and the CIQOL-10 Global. The CIQOL-35 Profile contains 35 items and measures functional abilities in six domains: Communication, Emotional, Entertainment, Environment, Listening Effort, and Social. The CIQOL-10 Global is a short form with 10 items drawn from the six domains that provide an overall assessment of abilities (McRackan et al., 2019b). When completing the instrument, CI users respond based on their endorsement of a particular statement in their daily life on a Likert scale that ranges from never to always. Reliability and validity of the instruments were confirmed in a study with a sample of *n* = 334 adult CI users from across the United States (Cronbach's α for all subscales = 0.84–0.92, test–retest reliability *r* = 0.83–0.90) (McRackan et al., 2021). That study found the CIQOL instruments to have superior psychometric properties compared to existing questionnaires (i.e., NCIQ: Cronbach's α was only applicable for two subscales = 0.89–0.90, test–retest reliability *r* = 0.77–0.92. HUI-3: Cronbach's α was not applicable, test–retest reliability *r* = 0.43–0.60).

**Table 1.** CIQOL Instrument Development.

Step	Content	Reference
Focus groups and item pool development	Focus groups with CI users Development of initial item pool (101 questions)	McRackan et al. (2017)
Item bank development	Online survey of the item pool Psychometric analysis resulting in item bank (81 items)	McRackan et al. (2019c)
Development of CIQOL instruments	Analysis of item bank using item response theory Item selection for profile instrument (35 items) and global measure (10 items)	McRackan et al. (2019b)
Validation of CIQOL instruments	Analysis for reliability and validity of the CIQOL-35 Profile and CIQOL-10 Global	McRackan et al. (2021)
Adaptation into German	Adaptation following guideline with forward translations, backward translations, and expert committee Test for comprehensibility in interviews with CI users	Pützer et al. (2024)
Validation of the German CIQOL instruments	Analysis for reliability and validity of the German adaptation	Present study

According to current German guidelines on cochlear implantation, it is essential to assess the quality of life of CI patients for treatment evaluation (Deutsche Gesellschaft für Hals-Nasen-Ohren-Heilkunde, Kopf- und Hals-Chirurgie e.V. [DGHNO-KHC], 2020, 2021). Therefore, making the CIQOL instruments available for clinicians and researchers who work with German-speaking CI patients would enhance CI services and research. For this purpose, the CIQOL instruments were translated and cross-culturally adapted into German language in a previous study (Pützer et al., 2024).

### Present Study

To confirm reliability and validity of the adapted German version, the present study reports on the validation of the German CIQOL instruments. We analyzed various psychometric properties of the German CIQOL instruments, such as internal consistency, convergent validity to an established quality of life instrument, and construct validity through factor analysis. The purpose of the present study was to evaluate whether the thorough adaptation process preserved the reliability and validity of the original instrument in its German version.

## Methods

### Study Design

Data for this study was collected through an online questionnaire. The questionnaire was created and administered through SoSci Survey, a secure web-based application for online questionnaires which is compliant with German data protection regulations. Participants completed the questionnaire anonymously. Collection of contact information was not necessary because the questionnaire was only completed at one time. Addresses were collected separately to other data for participants who chose to receive a small gift as an incentive for completing the questionnaire.

Participants were recruited through distribution of flyers electronically and on paper through German CI centers, rehabilitation clinics, and CI associations. Potential participants were led to the university project website to find further information about the project and a weblink directing to the online questionnaire. To be eligible for the study, participants were required to be at least 18 years old and have a bilateral hearing loss. As the CIQOL is intended to be administered in pre- and postoperative settings, having a CI was not a requirement for participation. Respondents were able to participate with CIs or hearing aids from any device manufacturer. Individuals with unilateral hearing loss and normal hearing in one ear were excluded for this study, to be consistent with the studies for the development of the CIQOL instruments in the English language.

### Measures

The questionnaire consisted of three major sections: (a) participant demographics, (b) the CIQOL-35 Profile, and (c) the NCIQ. Participant demographics comprised of questions about the personal background and situation, hearing loss, and CI characteristics (if applicable). Personal data were collected about age, sex, languages, education, employment, marital status, household size, residential area, and additional diseases or disabilities. Subjective social status was quantified with the MacArthur Scale (Adler et al., 2000). Participants were asked to rate their social status compared to other people in their country on a scale of one to ten, visualized as a ladder where people with the most money, most education and most respected jobs would be at the top. Hearing loss characteristics included the self-reported use of hearing devices, duration of hearing loss, tinnitus, and vertigo. For CI users, additional questions were asked about the duration of (first) CI, device manufacturer, reimplantations, daily wearing time and use of hybrid CI, which uses a shorter electrode array combined with acoustic amplification. On average participants took 18.75 min to complete the online survey.

The CIQOL-35 Profile consists of 35 statements about feelings and experiences in everyday situations. Participants are asked to rate these items on a 5-point scale from “Never” to “Always.” Items are grouped into the six domains Communication, Emotional, Entertainment, Environment, Listening Effort, and Social. After recoding reverse coded items and assigning the five answer categories to a numeric value of 1 to 5, a sum for each domain is calculated. These raw scores are transferred into interval scale outcome measures by using conversion tables (MUSC Health), resulting in outcomes ranging from 0 (*low functional ability*) to 100 (*high functional ability*). In addition to the outcome measures for the domains, a global measure is calculated with 10 selected items that are used for the short version of the instrument (CIQOL-10 Global; McRackan et al., 2019b). The 10 items for the CIQOL-10 Global score were selected by item response theory, including at least one item of each domain (McRackan et al., 2019b). For psychometric reasons, it is not possible to calculate a total score directly from the CIQOL-35 Profile, because its multiple unidimensional constructs and its domain scores do not reflect a single common latent trait.

The NCIQ was chosen as convergent validity criterion because it is the most cited CI-specific PROM and the instrument that is listed in the German guidelines for cochlear implantation (Deutsche Gesellschaft für Hals-Nasen-Ohren-Heilkunde, Kopf- und Hals-Chirurgie e.V [DGHNO-KHC], 2020). The NCIQ consists of 60 questions about the “CI situation” (Hinderink et al., 2000). Items are divided into three domains and six subdomains. The Physical domain entails the subdomains Basic sound perception, Advanced sound perception, and Speech production. The Psychological domain entails only the subdomain Self-esteem and the Social domain entails the subdomains Activity limitation and Social interactions. Participants are asked to answer the questions on a 5-point scale from “Never” to “Always” or from “No” to “Quite Well.” Additionally, items can be selected as “not applicable.” Scores were calculated following the NCIQ code book as described in Hinderink et al. (2000; first published online in 2016) and its corrigendum (Hinderink et al., 2017). Reverse coded items were recoded and answer categories were assigned to a value from 0 to 100 and then averaged for each domain, subdomain, and the total score. Although the code book allows a total score to be calculated, this has subsequently been found to not be psychometrically valid using modern measurement theory (McRackan et al., 2021). However, to follow the code book and to comply with common practice, we calculated total scores. Recently, a team of German researchers constructed a short version by selecting 25 items with the best uncorrected item-total correlation (Weichbold et al., 2024). We were able to calculate scores for the short version with our data, which were collected prior to the publication of the NCIQ short version. According to the code book participants were excluded if more than three items in a subdomain were not complete—that is if participants selected “not applicable” or did not give an answer. Because the German version of the NCIQ is not a public document, it was kindly provided to us by the developing team (i.e., Prof. Olze and her team; Charité—Berlin University Medicine).

### Data Analysis

The reliability of the instruments was tested through internal consistency and item analysis. These methods minimize efforts for the participants and simplify recruitment, as they offer robust results even if the questionnaire is filled out at only one point in time. Validity was assessed through content validity, convergent validity, and construct validity (Mokkink et al., 2010).

#### Internal Consistency

High internal consistency indicates that items on a scale measure the same latent construct. To evaluate internal consistency, we calculated *Cronbach's* α. Cronbach's α was applied separately to the different subscales. Additionally, Cronbach's α was calculated for the global outcome of the questionnaire (CIQOL-10 Global). We calculated *Cronbach's* α *if Item is Deleted* to determine if all items are valuable for the scale. Values of *Cronbach's* α *if Item is Deleted* show the change in Cronbach's α if a particular item was deleted. If the deletion of an item substantially increases the value of Cronbach's α, the reliability of the scale could be improved by deletion of that item (Field, 2009).

#### Item Analysis

We analyzed various aspects on an item level to evaluate the contribution of each item to the instrument. Values of the *Corrected Item-Total Correlation* show correlations between each item and the total score of the questionnaire to assess how well a single test item distinguishes between participants with high and low scores *(item selectivity)*. Scores on items with high item selectivity predict the result for the whole scale very well. Correlations above 0.32 assure that the item has at least 10% of common variance with the scale (Field, 2009). *Item difficulty* represents the likelihood that the participants check the best possible score on the item as calculated by dividing the mean score on the item by the highest possible score. Lower values indicate more difficult items, and higher values indicate easier items. To effectively differentiate between participants, difficulty indices should spread over the entire range of the measured ability, preferably between 20% and 80% (Döring, 2023). *Floor and ceiling effects* present the percentage of participants who obtained the highest or lowest possible score for the scale. If many participants check the highest or the lowest possible score on the scale, items might be too easy or too difficult (Döring, 2023). Floor and ceiling effects of less than 15% are preferred. By assuring that the instrument and each subscale include items with varying difficulty levels, we can ensure that the instrument accurately measures a range of levels of quality of life with CI.

#### Content Validity

To establish content validity, we looked for evidence that the content of the CIQOL instruments corresponds to the content of hearing-related quality of life measures. We followed Consensus-based Standards for the selection of health Measurement Instruments (COSMIN) methodology for assessing the content validity of PROMs (Mokkink et al., 2018). Following this guideline, we assessed relevance of the items and response options, comprehensiveness, and comprehensibility. This was achieved through a review of the development of the original instrument and the development of the German version.

#### Convergent Validity

Convergent validity was used to determine if the CIQOL instruments correlate with other established measures for hearing-related quality of life. We calculated correlations between scores for the CIQOL-10 Global with the full and short versions of the NCIQ. We additionally correlated domains of the CIQOL-35 Profile and scores of conceptually similar domains of the NCIQ to examine how the scores were associated with each other. Table 6 shows the conceptually similar components of the CIQOL instruments and the NCIQ. Not all subdomains of the CIQOL reflect a subdomain of the NCIQ.

The CIQOL domain *Communication* provides information regarding receptive and expressive communicative ability (McRackan et al., 2019b) and was compared to the NCIQ domain *Physical* functioning including the subdomains *Basic* and *Advanced sound perception* and *Speech production*. Items of the CIQOL domain *Emotional* evaluate the CI users’ emotional well-being and were compared to the NCIQ domain *Psychological* functioning, which is equal to the subdomain *Self-esteem*. The CIQOL domain *Entertainment* includes items about enjoyment and clarity of TV, music, and radio. The NCIQ only includes one item about television in the *Activity limitation* domain and a few items about listening to music in the *Advanced sound perception* domain, so there was no comparable subdomain for the CIQOL domain *Entertainment*. Items of the CIQOL domain *Environment* capture information about the ability to distinguish and localize sounds in the environment, which shows similarities to the NCIQ subdomain *Basic sound perception*. The CIQOL domain *Listening Effort* asks about CI users’ effort and fatigue associated with listening, which is not covered as a domain in the NCIQ. The CIQOL domain *Social* provides information about the ability to interact in groups and attend and enjoy social functions and is compared to the NCIQ domain *Social* with the subdomains *Activity limitation* and *Social interactions*.

We used nonparametric analysis and reported the correlation coefficient with Spearman Rho (Schober et al., 2018) as the two questionnaires are answered on a scale from “Never” to “Always.” This is because a linear correlation cannot be assumed a priori, and therefore, Pearson correlations might not identify all relevant correlations. For consistency with other publications on this topic, we also calculated Pearson's correlations. As the results showed similar patterns, we only reported the nonparametric results in this article. However, the parametric results with a comparison to the results of the validation of the original instrument (McRackan et al., 2021) are available in the Supplementary Material (Supplement 1). Correlations ≥0.70 were rated as strong, 0.50–0.69 as moderate, and <0.50 as weak (Rodgers & Nicewander, 1988).

#### Construct Validity

We evaluated the construct validity of the CIQOL instruments and the NCIQ with Confirmatory Factor Analysis (CFA; Brown, 2023) to test whether the structure of the questionnaire with its domains can be confirmed within the present data. We used the package “lavaan” in the statistical software R to conduct CFA with the diagonal weighted least squares (DWLS) estimation method. Results of the CFA were interpreted with the following pre-defined indicators of good model fit: (a) Root Mean Square Error of Approximation (RMSEA) ≤ 0.06 for good and ≤0.08 for adequate model fit, (b) Comparative Fit Index and Tucker-Lewis Index ≥0.95; and (c) Standardized Root Mean Square Residual (SRMR) ≤ 0.08 (Hu & Bentler, 1999), because it is important to consider fit indices from multiple categories (Brown, 2015). To evaluate the relationship between item and latent factor we investigated the Standardized Factor Loadings. The correlations among the latent variables give insights into the relations among the domains of the instrument. A minimum Standardized Factor Loading of ≥|0.32| (Tabachnick & Fidell, 2013) indicates a significant relationship between an item and the latent construct.

## Results

Two hundred and twenty-seven participants completed at least parts of the online questionnaire. Participants were automatically excluded by the system if they did not consent to the conditions of participation (*n* = 4), were younger than 18 years old (*n* = 1), did not complete more than 50% of the questions (*n* = 1), or had normal hearing in at least one ear (*n* = 13). Additional participants were excluded if not all items of the CIQOL instrument were completed (*n* = 1) or if there were more than three missing items per subscale in the NCIQ (*n* = 3). The data of the remaining 204 participants were included in the following analyses.

### Sample

The sample consisted of 204 participants aged between 19 and 87 years, the mean age was 53 years (Table 2). About 65% identified as female, 34% as male, and 0.5% as nonbinary. German was a native language for 98%. More than half of the sample was working and a third was retired. About 25% lived by themselves while half of the sample lived with one person and 27% lived with more than one person. About 16% had minors under the age of 18 living in their household. Most participants had their residence in Germany, only 1.5% outside of Germany. We were able to include at least one participant from 14 of the 16 different federal states in Germany. The distribution to the different states varied. With more than 50%, most participants lived in North Rhine-Westphalia, about 11% in Baden-Württemberg and 8% in Berlin. Additional medical conditions such as chronic diseases or disabilities were reported by a third of the sample. About half of the sample had tinnitus and almost a third had vertigo.

**Table 2.** Demographic Characteristics of the Study Sample (*n* = 204).

Variable	*n* (%)
Sex	
Female	133 (65.2)
Male	70 (34.4)
Non-binary	1 (0.5)
First language	
German	197 (96.6)
Bilingual-German	3 (1.5)
Other	4 (2.0)
School education	
Without degree	1 (0.5)
Main school or Middle school (9–10 years)	89 (43.6)
High school diploma (12–13 years)	108 (52.9)
Other	6 (2.9)
Professional education (multiple responses allowed)	
Without professional qualification	8 (3.9)
Still in studies/training	18 (8.8)
Completed vocational training	94 (46.1)
State-certified technician/master craftsman	21 (10.3)
University of applied sciences degree	32 (15.7)
University degree	51 (25.0)
Other	4 (2.0)
Employment	
Employed, full-time	71 (34.8)
Employed, part-time	40 (19.6)
Retired	68 (33.3)
Unemployed, seeing work	3 (1.5)
Unemployed, not seeking work	10 (4.9)
Unable to work	9 (4.4)
On parental leave	3 (1.5)
Marital status	
Not married / no domestic partnership	52 (25.5)
Married / domestic partnership	123 (60.3)
Divorced / separated	15 (7.4)
Widowed	14 (6.9)
Size of household	
Live by themselves	51 (25.0)
With one person	97 (47.5)
With two people	33 (16.2)
With three people	20 (9.8)
With more than three people	3 (1.5)
Minors (<18 years) in household	
No children	170 (83.3)
One child	25 (12.3)
Two or three children	8 (3.9)
Not answered	1 (0.5)
Residential setting	
Urban	113 (55.4)
Suburban	40 (19.6)
Rural	51 (25.0)
Country of residence	
Germany	201 (98.5)
Other (Austria, Luxemburg, Spain)	3 (1.5)
State of residence	
Baden-Württemberg	22 (10.8)
Bavaria	7 (3.4)
Berlin	17 (8.3)
Brandenburg	8 (3.9)
Bremen	1 (0.5)
Hesse	11 (5.4)
Mecklenburg-Western Pomerania	2 (1.0)
Lower Saxony	9 (4.4)
North Rhine-Westphalia	109 (53.4)
Rhineland-Palatinate	5 (2.5)
Saxony	2 (1.0)
Saxony-Anhalt	2 (1.0)
Schleswig-Holstein	4 (2.0)
Thuringia	2 (1.0)
Other (Luxemburg, Vienna)	2 (1.0)
Not answered	1 (0.5)
Subjective social status (MacArthur Scale)	
1–2 (Low)	3 (1.5)
3–4	12 (5.9)
5–6	49 (24.0)
7–8	102 (50.0)
9–10 (High)	36 (17.6)
Missing data	2 (1.0)
Medical conditions related to the hearing system (multiple responses allowed)	
Tinnitus	103 (50.5)
Vertigo	56 (27.5)
No Tinnitus or Vertigo	83 (40.7)
Additional medical conditions	
Yes	71 (34.8)
No	133 (65.2)

### Hearing Loss and Hearing Device Characteristics

Per inclusion criteria we excluded participants who reported to have normal hearing in at least one ear. The duration of hearing loss in the sample ranged between less than a year to more than 30 years or congenital hearing loss (Table 3). While a third had had hearing loss for more than 30 years and another 23% had been born with hearing loss, only 44% reported the onset of their hearing loss sometime in the last 30 years. The sample consisted of participants with bilateral CIs (57%), with one CI and one hearing aid (33%), with one CI and no device in the other ear (8%), and with bilateral hearing aids (2%). About 200 participants reported the use of at least one CI. Information on CI characteristics from one participant were excluded because they stated to have bilateral hearing aids and the date of the implantation was in the future, suggesting the person was a CI candidate but did not yet have a CI. For another two participants only the duration of CI use was excluded because it was not plausible that the implantation would have been in the years 1918 or 2029. Participants reported having received their first CI between 1987 and 2022 (196 respondents). The duration of CI use at the time of survey was between 2 months and 35 years, 6 months with a mean of 8 years, and 10 months. The participants stated that they wore their CI on average for 14.5 h a day, the majority reported a wearing time between 12 and 20 h. The distribution regarding manufacturers varied between the four CI manufacturers Advanced Bionics, Cochlear, MED-EL, and Oticon. About 8% of the sample reported that they had had reimplantation and 7% had a hybrid CI.

**Table 3.** Hearing Loss and Hearing Device Characteristics of the Study Sample.

Variable	*n* (%)	
*Hearing loss and hearing devices (n = 204)*		
Duration of hearing loss		
Less than 1 year	3 (1.5)	
1–5 years	13 (6.4)	
5–10 years	19 (9.3)	
10–20 years	28 (13.7)	
20–30 years	27 (13.2)	
More than 30 years	66 (32.4)	
Congenital	48 (23.5)	
Listening modality		
Bilateral CI	116 (56.9)	
Bilateral hearing aid	4 (2.0)	
Bimodal CI with contralateral hearing aid	68 (33.3)	
Unilateral CI with no contralateral device	16 (7.8)	
Hearing device side	Left	Right
Cochlear Implant	157 (77.0)	159 (77.9)
Hearing aid	39 (19.1)	37 (18.1)
No device	8 (3.9)	8 (3.9)
*Cochlear implant (CI) characteristics (n = 200)*		
Duration of (first) CI		
Less than 1 year (0;0–0;11)	23 (11.5)	
1–5 years (1;0–4;11)	56 (28.0)	
5–10 years (5;0–9;11)	48 (24.0)	
10–20 years (10;0–19;11)	43 (21.5)	
20–30 years (20;0–29;11)	25 (12.5)	
More than 30 years (>30;0)	1 (0.5)	
Missing data	4 (2.0)	
CI company (multiple responses allowed)		
Advanced Bionics	28 (14.0)	
Cochlear	117 (58.5)	
MED-EL	55 (27.5)	
Oticon	2 (1.0)	
Reimplantation		
No	184 (92.0)	
Yes	16 (8.0)	
Hybrid CI/Electric Acoustic Stimulation		
Yes	15 (7.5)	
No	179 (89.5)	
I don’t know	6 (3.0)	
Wearing time per day		
Less than 4 h	3 (1.5)	
4 to 8 h	3 (1.5)	
8 to 12 h	17 (8.5)	
12 to 16 h	102 (51.0)	
16 to 20 h	73 (36.5)	
More than 20 h	1 (0.5)	

### Missing Data

As mentioned above, only one participant was excluded because of one missing item of the CIQOL and three participants were excluded because they had more than three missing items per subscale in the NCIQ. In the included sample of 204 participants, there were still 269 missing responses (2.2%) in the 60 items of the NCIQ. For 80% of items, no more than 2% of data were missing. However, some items stood out because many participants chose not to answer them (Table 4). For example, a third of the participants did not answer the question on the NCIQ whether their hearing impairment presents a problem in the contact with deaf persons. The most frequently not completed items asked the participants about their voice/speech, contact with deaf people, work/studies, and people they live with.

**Table 4.** Items With the Highest Missing Response Rates for the NCIQ.

Item No.	Item of the NCIQ	Missing Responses (in%)
Item 8	Does your hearing impairment present a serious problem in your contact with **deaf persons**? (Social interactions)	33.3
Item 39	How often does it annoy you that persons can hear from **your voice/speech** that you have a hearing problem? (Self-esteem)	17.2
Item 6	Does your hearing impairment present a serious problem during your **work or studies**? (Activity limitations)	13.2
Item 27	Can strangers hear from **your voice** that you are deaf or hearing-impaired? (Speech production)	6.4
Item 55	Does your hearing impairment prevent you from sticking up for yourself (at work, in relationships)? (Activity limitations)	5.4
Item 59	Can you make **your voice** sound “natural” (so that is does, not sound like a deaf person's voice)? (Speech production)	4.4
Item 20	Does your hearing impairment present a serious problem in your contact with the **persons you live with** (family/partner)? (Social interactions)	4.4

*Note.* Key aspects of the items that may be related to missing responses are highlighted in bold.

### Reliability

We used internal consistency and item analysis to test the German CIQOL instruments for reliability (Table 5). Item analysis included item selectivity, item difficulty, and floor and ceiling effects.

#### Internal Consistency

Our analysis showed good internal consistency of the German CIQOL-35 Profile in the different subdomains and the global score with *Cronbach*‘*s* α of 0.84 to 0.91 (Table 5) (Field, 2009). Overall, the results for *Cronbach's* α *if Item Deleted* were lower than Cronbach‘s α for each scale, which means that the items do not cause a substantial decrease in α. There was only one item that would improve Cronbach's α for the Entertainment scale for about 0.007 from 0.889 to 0.896 if delete—which is a negligible amount (Item 16).

**Table 5.** Reliability and Item Analysis for the CIQOL Instruments.

CIQOL Domain	Cronbach's α	Cronbach's α If Item Deleted (Range)	Item Selectivity (Range)	Item Difficulty (Mean, Range in%)	Floor Effect	Ceiling Effect
*Indication of reliability*	*>*.*8*	*<Cronbach's* α	*>.32*	*20%–80%*	*<15%*	*<15%*
Communication	.907	.892–.906	.516–.756	55, 29–81	0%	0%
Emotional	.844	.788–.827	.602–.734	61, 42–72	0%	2.5%
Entertainment	.889	.843–**.896**	.584–.820	63, 57–72	1%	6.4%
Environment	.866	.812–.860	.606–.804	72, 50–81	0%	2.5%
Listening Effort	.857	.813–.841	.623–.728	42, 18–62	1.5%	0%
Social	.880	.828–.877	.634–.820	73, 61–81	0%	8.3%
CIQOL-10	.873	.847–.868	.504–.767	58, 18–81	0%	0%

*Note.* Results for Cronbach's α if Item Deleted that exceed Cronbach‘s α for the scale indicating possible problems with an item are highlighted in bold.

#### Item Analysis

*Corrected Item-Total Correlations* between each item and the total score of each CIQOL-35 Profile domain with 0.52–0.82 were substantially higher than the required minimum of 0.32 (Table 5). Item selectivity for the global outcome measure was also very good (>0.50), even though the instrument measures a multidimensional construct. *Item difficulty* for each item in the instrument ranged between 18% and 81% with a mean of 60%. The domain *Listening Effort* included the most difficult items with a mean of 42% and the domains *Social* and *Environment* included the easiest items with a mean of 73% and 72%. There were no substantial floor or ceiling effects for the different domains. No participant reached the best or worst possible score on the questionnaire. About 1.5% of participants scored the lowest possible score on the domain *Listening Effort* and 1% on the domain *Entertainment*. For all other domains, no one received the lowest possible score. On the domain *Social* 8.3% of participants showed a ceiling effect, on the domain *Entertainment* 6.4% and on the domains *Emotional* and *Environment* 2.5% each. In the other two domains, none of the participants scored the highest possible score.

### Validity

To establish validity of the German CIQOL instruments, we analyzed content validity, convergent validity and construct validity. Content validity for this PROM was evaluated following COSMIN methodology. The NCIQ served as an external measure for assessing convergent validity. We compared the psychometric properties related to construct validity of the newly adapted CIQOL instruments with those of the widely used NCIQ.

#### Content Validity

The development process of both the original and the German CIQOL instruments (Table 1) accounted for the relevance, comprehensiveness, and comprehensibility of the questionnaire, thereby ensuring high content validity for the German CIQOL versions (Mokkink et al., 2018). The original instruments were developed following recent guidelines for developing PROMs, including a participatory research design. To ensure the relevance of the included items for the target population, the contents for the original instruments were identified through focus groups representing the target population of adult CI users (McRackan et al., 2017). Participants in that earlier study had the opportunity to identify (new) important topics that affect their lives and to confirm or deny topics that were used in prior measures. Based on the results of the focus groups and a comprehensive literature review, an item bank and associated domains were developed. The final original instruments were developed through an analysis of the item bank with item response theory (McRackan et al., 2019b).

The development of the German version of the CIQOL instruments aimed to preserve the established relevance and comprehensiveness of the original version by following a practice guide for translating and adapting hearing-related questionnaires (Hall et al., 2018). Through multiple forward and backward translations, a review through an expert committee and pilot testing of the instrument in interviews with CI users, the authors ensured the comprehensibility of the questionnaire in German language and culture, while maintaining equivalence to the original version (Pützer et al., 2024). The comprehensibility of the adapted PROM was assessed through cognitive interviews with a sample representing the target population. About 15 adult CI users assessed the instruments’ instructions, items, and response options to confirm that they have the intended meaning. Any phrases and items that were perceived as difficult were revised to improve comprehensibility.

#### Convergent Validity

We found significant positive correlations between several domains and the Global outcome of the German CIQOL, with the NCIQ, confirming the convergent validity of the instrument (Table 6). The CIQOL-10 Global correlated strongly with both the total score (*r_s_* = 0.85) and the short version of the NCIQ (*r_s_* = 0.83). Results for the NCIQ-Total were moderately to strongly correlated with all CIQOL domains. All higher-level domains of the NCIQ correlated strongly with the designated CIQOL domains. Some of the NCIQ subdomains showed only moderate correlations with the CIQOL domains: *NCIQ-Basic sound perception* and *NCIQ-Speech production* with *CIQOL-Communication*, and *NCIQ-Activity limitation* with *CIQOL-Social*. All correlations were statistically significant with *p * < .01. Accordingly, this study suggests that the CIQOL instruments measure a similar construct as the NCIQ. The dedicated subscales also measure similar constructs. However, there were no matching domains in the NCIQ instrument for the CIQOL domains *Entertainment* and *Listening Effort*. The NCIQ subdomain *Basic sound perception* from the *Physical* domain showed a stronger correlation to the CIQOL domain *Environment* than to the CIQOL domain *Communication*.

**Table 6.** Convergent Validity for the CIQOL and NCIQ Instruments.

CIQOL Domain	NCIQ Domain/Subdomain	Spearman Rho (*r_s_*)
Communication	NCIQ Total	.**752**
	Physical	.**728**
	Basic sound perception	.598
	Advanced sound perception	.**757**
	Speech production	.552
Emotional	NCIQ Total	.**740**
	Psychological	.**796**
	Self-esteem	.**796**
Entertainment	NCIQ Total	.665
Environment	NCIQ Total	.**724**
	Basic sound perception	.**750**
Listening Effort	NCIQ Total	.**725**
Social	NCIQ Total	.**757**
	Social	.**740**
	Activity limitation	.683
	Social interaction	.**742**
CIQOL-10 Global	NCIQ Total	.**848**
CIQOL-10 Global	NCIQ Short version	.**825**

*Note.* Strong correlations are highlighted in bold.

#### Construct Validity

The results of the CFA are shown in Table 7. Most a priori established indices of model fit (Hu & Bentler, 1999) indicated good model fit for the CIQOL-35 Profile, the CIQOL-10 Global and the six domains. RMSEA indicated poor model fit for all CIQOL-35 domains but met the threshold for adequate model fit for the CIQOL-35 Profile and CIQOL-10 Global. This may be because RMSEA underestimates fit at small sample sizes of *n* < 200 (Brown, 2015; West et al., 2023) and with small degrees of freedom (Kenny et al., 2015). The latter may explain why the only two models with adequate model fit were those with larger degrees of freedom. The *Entertainment* and *Environment* domain did not show acceptable SRMR, but the results (0.085, 0.088) were close to our cut-off threshold of 0.08 and CFI and TLI indicate good model fit. All items had standardized factor loadings of >0.6 on their domains.

**Table 7.** Confirmatory Factor Analysis: Model Fit and Standardized Factor Loadings for the CIQOL and NCIQ Instruments.

Instrument Domain	Test Statistic: Chi Square	df	RMSEA	CFI	TLI	SRMR	SFL (Min–Max)
*Cut-off thresholds for good model fit*			*<.06*	*>.95*	*>.95*	≤*.08*	≥*.32*
German Cochlear Implant Quality of Life (CIQOL)-35 Profile							
CIQOL-35 Profile	1192.210	545	.076	.**988**	.**989**	.**072**	**.645–.949**
Communication	171.184	35	.138	.**984**	.**980**	.**078**	**.610–.881**
Emotional	14.304	5	.096	.**995**	.**989**	.**047**	**.694–.901**
Entertainment	58.624	5	.230	.**990**	.**981**	.085	**.756–.942**
Environment	48.478	5	.207	.**990**	.**980**	.088	**.716–.981**
Listening Effort	24.820	5	.104	.**992**	.**983**	.**065**	**.737–.890**
Social	11.769	5	.082	.**998**	.**997**	.**042**	**.723–.968**
CIQOL-10 Global	80.537	35	.080	.**988**	.**985**	.**071**	**.619–.886**
German Nijmegen Cochlear Implant Questionnaire (NCIQ)							
NCIQ Total	3340.247	1695	.069	.**984**	.**984**	.**078**	.002–.882
Physical	1845.642	405	.132	.947	.973	.111	**.405–.851**
Basic sound perception	66.113	35	.066	.**994**	.**992**	.**057**	**.521–.846**
Advanced sound perception	41.062	35	.**029**	.**998**	.**998**	.**045**	**.544–.789**
Speech production	184.115	35	.145	.**978**	.**971**	.102	**.534–.941**
Psychological	75.919	45	.076	.**993**	.**992**	.**053**	.113–.858
Self-esteem	75.919	45	.076	.**993**	.**992**	.**053**	.113–.858
Social	313.617	170	.065	.**993**	.**992**	.**066**	**.362–.875**
Activity limitations	41.062	35	.**029**	.**998**	.**998**	.**045**	**.536–.810**
Social interactions	3229.651	45	.590	.641	.561	.247	.305–.868
NCIQ short version	1016.289	275	.115	.**979**	.**977**	.087	.304–.852

*Note.* Values meeting the a priori established criteria for good model fit (Hu & Bentler, 1999) are highlighted in bold, values for adequate model fit are underlined. RMSEA indicates root mean square error of approximation; CFI indicates comparative fit index; TLI indicates Tucker-Lewis index; SRMR indicates standardized root mean square residual. SFL indicates Standardized factor loadings of the variable for the latent factor.

For the NCIQ, the fit indices for the domains *Physical* and *Social interactions* did not meet any thresholds for good model fit. For the *NCIQ short version* and the domain *Speech production*, only CFI and TLI met the cut-off threshold. The domains *Basic sound perception*, *Advanced sound perception*, *Psychological/Self-esteem*, *Social*, and *Activity limitations*, as well as the full version of the questionnaire, however, showed adequate to good model fit in RMSEA and good model fit for the other fit indices. Overall, standardized factor loadings of the observed variables on the latent factors were lower than for the CIQOL-35 Profile. Two items did not meet the minimum threshold of 0.32, which means that they share less than 10% of their variance with the latent factor (Items 8 and 28).

Covariances between the latent factors of the CIQOL-35 Profile are shown in Table 8. All latent factors correlated moderately to strongly with each other. The strongest relationships were found between the domains *Listening Effort* and *Communication* (0.91) and the domains *Emotional* and *Social* (0.81). The domain *Listening Effort* was strongly correlated with four of the other five subscales: *Communication*, *Emotional*, *Environment*, and *Social*.

**Table 8.** Covariances Between the CIQOL Domains.

Latent Variables	Communication	Emotional	Entertainment	Environment	Listening Effort	Social
Communication						
Emotional	.675					
Entertainment	.619	.567				
Environment	**.** **752**	.640	.688			
Listening Effort	.**913**	.**707**	.601	.**711**		
Social	.634	.**809**	.531	.596	**.740**	

*Note.* Strong correlations (≥.70) are highlighted in bold.

## Discussion

Various reported measures confirmed reliability and validity of the German versions of the CIQOL instruments. Internal consistency and item analysis showed that the domains and the global score measure a single construct and that the items within each domain are highly discriminative. The content selected for the CIQOL instruments appears valid, the structure is meaningful, and the instruments measure a construct comparable to that of an established CI quality of life instrument.

### Reliability

Internal consistency and item analysis confirmed the reliability of the instruments. All subscales of the CIQOL-35 Profile and the CIQOL-10 Global showed good internal consistency with a Cronbach's α >0.8, indicating that items on the domains measure the same latent construct. There would not be a relevant benefit for the questionnaire or the subscales if any item were deleted. All items correlated positively moderately or strongly with their respective domain and the global outcome, indicating that they do represent the underlying trait they are meant to represent. Item difficulty ranged between 18% and 81% indicating that the entire range of the measured trait is covered. We can assume that the items are neither too easy nor too difficult because the subscales did not show critical ceiling or floor effects. We saw the highest percentage of people who reached the best possible score on the domains Social (8.3%) and Entertainment (6.4%). That means some participants did not perceive any difficulties in social contacts or in the enjoyment of entertainment media in their everyday life. The lowest score on any domain or the global outcome was rarely observed. Previous studies showed that quality of life improves for CI candidates after cochlear implantation. Considering that there were very few pre-CI participants in our sample, the questionnaire would be able to capture results for CI users or CI candidates with lower quality of life experiences.

### Content Validity

The COSMIN criteria for content validity: relevance, comprehensiveness, and comprehensibility (Mokkink et al., 2018) point to a good content validity for the German CIQOL instruments. To evaluate these criteria, we assessed the development of the original instruments and the German version (Table 1). The thorough process of developing the CIQOL instruments as reported previously (McRackan et al., 2017) ensured that the content of the questionnaire aligns with relevant aspects of quality of life for CI users that are impacted by cochlear implantation. Modern PROM development standards (e.g., COSMIN) require direct engagement with the target population to ensure that domains reflect what patients consider important. The need to examine and revise ICF Core Sets through patient focus groups has already been recognized in the ICF framework (Stucki & Grimby, 2004). A limiting factor is the sample that was used for the focus groups when developing the original English version of the CIQOL. People with single-sided deafness were not represented in the sample and might experience different changes in quality of life after cochlear implantation. Therefore, the content of the instrument is expected to be representative of the population of adult CI users with bilateral hearing loss.

Overall, the contents that were derived from the focus groups align with current research topics and PROMs for the CI population. The impact of hearing loss and cochlear implantation on a range of aspects of quality of life, such as conversation, family relationships, and community life, has been documented in previous studies (Andries et al., 2024). Other studies focus on the impact of CI on selected quality of life related aspects, such as listening effort and fatigue (Philips et al., 2023), social outcomes (Bekele Okuba et al., 2023), or music (Bleckly et al., 2024).

During the adaptation of the questionnaire into German, no alterations were made regarding the items or domains of the instrument (Pützer et al., 2024). The adaptation ensured that the contents of the German version are equivalent to the original. Comprehensibility of the German instruments was confirmed through cognitive interviews. The evaluation of content validity in the present study confirms that the German CIQOL instruments contain relevant contents of hearing-related quality of life, are comprehensive, and comprehensible.

### Convergent Validity

We decided to use the NCIQ for convergent validity because at the time of planning this study it was one of few instruments that specifically addresses quality of life in adults with CI. The NCIQ is commonly used and available in various languages, including German. Correlations between the instruments prove the CIQOL and NCIQ measure the same construct. Similar subscales of both questionnaires also measure similar constructs. However, some domains of the NCIQ did not reflect in a domain of the CIQOL and some subdomains only correlated moderately with each other.

We did not find related domains in the NCIQ for the CIQOL domains *Entertainment* and *Listening Effort*, which are relevant constructs for a CI users’ quality of life as established by the patient focus groups used to develop the CIQOL instruments (McRackan et al., 2017). The NCIQ domain *Physical* correlated strongly with the CIQOL domain *Communication*. However, two of the NCIQ subdomains *Speech production* and *Basic sound perception* only correlated moderately with the CIQOL domain *Communication*. Items about the production of speech are not covered in the CIQOL-35 Profile instrument, which may explain the missing correlation with *NCIQ-Speech production*. This topic did not derive as relevant for CI users’ quality of life from the focus groups (McRackan et al., 2017). Examination of the NCIQ domain *Basic sound perception* showed that these items are more focused on environmental sounds than on communicative situations. This explains the weaker correlation with *CIQOL*-*Communication* and why we found a stronger correlation with the CIQOL domain *Environment*.

Overall, the adapted German version of the CIQOL measures a similar construct as the established German version of the NCIQ, even though the structure and domains of the instruments differ. Recent German CI guidelines suggest the NCIQ as an instrument to measure quality of life in CI users (Deutsche Gesellschaft für Hals-Nasen-Ohren-Heilkunde, Kopf- und Hals-Chirurgie e.V [DGHNO-KHC], 2021). Results of this study reveal the adapted German version of the CIQOL as a valid alternative that might even have some advantages over the NCIQ.

### Challenges with the NCIQ

The NCIQ has been criticized for its topicality, length (Lenarz et al., 2022), and the methodology used to develop the questionnaire (Andries et al., 2021). A major part of content validity of the CIQOL comes from the derivation of an item pool based on patient focus groups and a psychometric approach for item selection. Items for the NCIQ were selected by experts based on intuitive judgement and experiences (Hinderink et al., 2000). Numerous items were adapted either from published questionnaires or from informal item pools from patient rehabilitation. This approach harbors the risk of reproducing items that are not (or no longer) relevant and missing relevant topics for hearing related quality of life in adults with CI. Our results indicate that the contents of the NCIQ domain *Speech production* might not be relevant to CI users today, even though they may have been relevant at times with a different CI population. This could explain why some studies evaluating quality of life in CI users did not observe significant improvements in the *Speech production* subdomain (Knopke et al., 2016). Additionally, details about the development of the German version of the NCIQ are not published, neither is the German version itself. This is another advantage of the German version of the CIQOL, where the thoroughly reported cross-cultural adaptation supports the content validity of the instrument (Pützer et al., 2024).

In the present study, the response rate for the NCIQ items was lower than for the CIQOL-35 Profile items. One factor may be that the NCIQ was the second questionnaire in the online survey and participants may have been less motivated toward the end of the survey. Moreover, the NCIQ explicitly gives the option to answer items with “not applicable,” which may have resulted in participants using this option more frequently. To make sure that participants do not feel burdened by potentially outdated terms used in the NCIQ, participants were made aware that some items of the NCIQ may be no longer adequate but that it would still be very important for the study that they respond to as many items as possible. This could have influenced participants’ responses. Another reason for missing answers could be its considerable length of 60 items.

We noticed certain items with exceptionally high rates of missing data, possibly because participants did not find them applicable for their experiences. A third of our sample did not answer a question about their contact with deaf people (Item 8). Presumably this question means to ask about the contact to people using sign language, because the communication mode would differ from contact to people using spoken language. The reason that a majority of our sample did not find this question applicable could be that they do not have contact to people who are deaf. As most participants stated to have acquired hearing loss, it is likely that they grew up in a spoken language environment and are not necessarily connected to the Deaf community.

Three of the frequently unanswered items inquire about the person's ability to modify their voice and speech to “sound natural.” Apparently, this topic was not applicable for many participants. The items imply the concept of a certain “deaf person's voice” that should be avoided. If people do not perceive their voice as problematic these items are irrelevant. Cultural changes in the perception and acceptance of disabilities may have led to a more critical view on these items today. Additionally, improvements in CI technology and changes in indication criteria for CI from more than 25 years ago—when the NCIQ was developed—to today may have resulted in more CI users who do not perceive difficulties with their own voice pre or post CI. Being regularly in contact with deaf people and having problems hearing and modifying their own voice may have been more relevant for people with CI at earlier times in CI care. Nowadays these items might not be applicable for a majority of CI users.

Some other items that were frequently unanswered could still be relevant today. These missing data might be explained by the sample demographics. One question is about work, another question is about the contact to people one is living with, and another is about work and relationships. As 45% of our sample were not working due to various reasons and 25% stated they live by themselves, they may have stated that the questions were not applicable.

Overall, the factor structure for the CIQOL instruments is superior to the NCIQ instruments. More fit indices met the criteria for good model fit and the standardized factor loadings were higher for the CIQOL. This may relate to the stringent psychometric analyses and validation of the CIQOL instruments (McRackan et al., 2019b, 2019c, 2021). Fit indices for the NCIQ domains *Physical* and *Social interactions* did not indicate good model fit. For the three subdomains of the domain *Physical*, most fit indices pointed to good model fit. The results of the CFA indicate that the three subdomains lack a unifying latent construct. The ten items of the subdomain *Social interactions* do not have an underlying latent construct. This subdomain was constructed to evaluate the CI user's communication and contacts in various contexts. Items 8 and 32 of this subdomain had the smallest standardized factor loadings and shared only about 10% of their variance with the latent factor. We already established that item 8 might be problematic because it asks about contact with deaf persons. Item 32 “Do you go places where your hearing impairment might present a serious handicap?” differs from the other items on the subdomain, because it is not about a specific communicative situation or the contact to a specific group of people. In the domain *Psychological* and the identical subdomain *Self-esteem*, item 28 “Do you ask other persons to speak more loudly or clearly if they are speaking too softly or unclearly” had a low standardized factor loading. This item differs from other items in the domain because the question is not specific to a feeling or emotion (e.g., “Do you feel …”). The results of the CFA for the NCIQ indicate that the factor structure of the instrument could be improved if certain items were removed. In the validation study of the English CIQOL instruments, the CFA also gave poor results, limiting additional analysis for the NCIQ (McRackan et al., 2021).

Our findings support previous criticism of the NCIQ (Ambert-Dahan et al., 2018; Andries et al., 2021; Lenarz et al., 2022; McRackan et al., 2019c). The development of the items in the questionnaire and the adaptation into German lack participation of CI users, which decreases content validity. Some items seem to be outdated and do not apply to people with CI nowadays. The NCIQ validation study did not use factor analysis (Hinderink et al., 2000) and our results shows some problems with the factor structure, resulting in limited construct validity. Problems with the length of the questionnaire were addressed by the development of the short version (Weichbold et al., 2024); however, methodical and content-related difficulties were not taken into account.

### Construct Validity

The CFA proved the factor structure of the German version of the CIQOL instruments is valid, and the domains are well constructed. All items unidimensionally load on the dedicated latent factors that are represented as domains in the full version of the instrument. The model of the full instrument with its 35 items, six domains, and one superordinate latent construct (quality of life) also shows good model fit. Even though the Global version of the questionnaire is not divided into the different domains, CFA proves an underlying latent construct for those 10 items. Therefore, the CFA supports the construct validity of the German CIQOL instruments.

We observed strong covariances between some of the latent factors. Correlation between factors in a PROM is not uncommon because factors in a PROM are interrelated aspects of a broader experience, yet they remain separable when they have unique qualitative meaning and when CFA analyses support distinct item functioning. Here, we observed strong covariances between the factors *Communication* and *Listening Effort* as well as *Social* and *Emotional.* These relationships can be explained by the content of the domains. Items in the domain *Listening Effort* represent the effort and fatigue associated with listening. All five items inquire about the listening effort a person experiences when following a conversation in certain environments. The domain *Communication* focuses on the ability to participate and understand conversations in different situations. It stands to reason that an individual experiencing difficulties with listening effort would also perceive problems in communication, and vice versa. This may apply specifically to conversations in challenging communicative situations, for example, in groups or in noisy environments. The relationship between the domains *Social* and *Emotional* is not as transparent. Items in the domain *Emotional* address the impact of hearing ability on emotional well-being. The domain *Social* entails items about the ability to interact in groups and to attend and enjoy social functions. These domains may be intertwined through an interdependency. People who struggle with social situations because of their hearing loss may feel negative emotions about their psychological well-being, and at the same time, people who experience negative emotions because of their hearing loss may avoid social situations because they are not as confident or resilient.

*Listening Effort* seems to be the factor with the strongest correlations with the other factors. Four out of five domains correlate strongly with *Listening Effort*, indicating that the perceived effort when listening to conversations may influence a broad spectrum of experiences in everyday life. Our analyses demonstrated that *Listening Effort* items load strongly on their own factor and capture variation not adequately explained by *Communication* performance alone. In the foundational focus groups with CI users, listening effort consistently appeared as a unique and meaningful aspect of CI users’ lived experience (McRackan et al., 2017). The domain has strong face validity and content validity, as its inclusion was driven by CI users themselves. Thus, it is valuable to retain *Listening Effort* as a distinct domain.

Interestingly, listening effort has been found to be independent from correct responses in a speech recognition test, and depends on the necessity to mentally repair misperceived speech (Winn & Teece, 2021, 2022). Other questionnaires for quality of life or listening quality, for example, the NCIQ (Hinderink et al., 2000), HL-QOL (Illg et al., 2023), and SSQ (Gatehouse & Noble, 2004) do not explicitly evaluate listening effort. However, questionnaires are available that separately evaluate listening effort in more detail, for example, LEQ-CI21 (Hughes et al., 2021). Even if reliable objective indices of listening effort—which are already used in some contexts—were widely available, they would not replace the need for a patient reported domain capturing the subjective, experiential component of listening effort that the CIQOL domain evaluates.

### Cross-Language Equivalence

Similarities were found when comparing reliability and validity results for the German CIQOL instruments to the results of the English validation (McRackan et al., 2021). For reliability and item analysis, we were able to compare Cronbach's α and floor and ceiling effects as these were used in both studies. Results for Cronbach's α ranged between 0.84 and 0.92 (McRackan et al., 2021) for the original study, and 0.84 and 0.91 for this study (Table 5) for all domains. In both studies the domain *Communication* showed the highest α and *Emotional* and *Listening Effort* showed the lowest results. Floor and ceiling effects showed similar patterns as well and did not exceed the criterion of 15% for any domain. A ceiling and floor effect of 0% was observed for the CIQOL-10 Global and the domain *Communication* in both studies. The highest ceiling effects were documented for the domains *Social* of about 8% and *Entertainment* of about 6%. The only domains exceeding a floor effect of 0% were the domains *Entertainment*, *Environment* and *Listening Effort* in the English validation study (McRackan et al., 2021) whereas only *Entertainment* and *Listening Effort* in the present study. Overall reliability and item analysis results for both studies show a lot of equivalence.

Convergent validity was also determined through correlations with the NCIQ in the English and German validation studies. Moderate to strong correlations were found between conceptually similar domains and the total result of the NCIQ with all CIQOL-35 domains in both studies. The results were congruent, Pearson correlations differing between 0.00 and 0.13 (Supplement 1). The NCIQ domain *Speech production* did not have strong correlations with the CIQOL domain *Communication* in both studies. We were not able to compare correlations between *CIQOL-Environment* and *NCIQ-Basic sound perception* and correlations with the NCIQ short version, because these were not reported in the validation study of the original instrument (McRackan et al., 2021).

Results of CFA to assess the construct validity of the instrument were comparable in the validation studies. In both studies, all items had standardized factor loadings ≥ 0.32. CFI and TLI met a priori established criteria for good model fit for all domains and the global outcome. RMSEA did not meet the threshold for good model fit in most subscales in both validation studies, only for the domain *Communication* and the CIQOL-10 Global outcome in the English validation. SRMR values were slightly above the threshold for the domain *Entertainment* in both studies and additionally for the domain *Environment* in the German validation. Results indicate that the factor structure is equivalent across the two languages.

Availability of questionnaires in multiple languages has many advantages for use in research and clinical practice. Studies with the same instrument in different languages allow researchers to compare outcomes among countries and regions and to replicate research designs. It offers the possibility of evaluating a certain construct in a group of people with various languages, for example in transnational studies. It enables clinicians to engage people with a questionnaire in their preferred language and thereby reduces language barriers.

At the moment, the CIQOL instruments are available in nine languages. There is ongoing research with the CIQOL instruments to broaden clinical usability and impact, for example, with normative data (McRackan, Hand, Chidarala, Velozo & Dubno, 2022), a functional staging system (McRackan, Hand, Velozo & Dubno, 2022), and a CIQOL-35 Expectations instrument (McRackan, Hand, Chidarala & Dubno, 2022). A CIQOL Digital platform including an app and web interface will be released soon to improve scoring and interpretation to enhance clinical implementation. The instruments have already been used in several research studies, for example, about CI satisfaction and decisional regret (Shannon et al., 2023) or discrepancies between expected and actual CI-related outcomes (Fabie et al., 2023).

### Implications for Research and Practice

Confirming reliability and validity, this study demonstrates the German CIQOL instruments as valuable, ready to use tools in clinical practice and research with adults with CI. Indicators for reliability (e.g., Cronbach's α) and validity (e.g., fit indices and standardized factor loadings) were minimally superior for the CIQOL-35 Profile compared to the CIQOL-10 Global. Through displaying results for the domains, the CIQOL-35 Profile also gives a more comprehensive picture. This could have clinical implications for rehabilitation after cochlear implantation, monitoring changes in functional abilities over time, and for preoperative counseling. Measuring quality of life complements audiometric and speech recognition outcomes and provides a broader understanding of the impact of CI on a person's everyday life. This is highly relevant, since studies have shown that there are only weak correlations between audiometric or speech recognition outcomes and quality of life (McRackan et al., 2019a; Moberly et al., 2018).

Even though the participant information stated that the study aimed to recruit participants with bilateral hearing loss, 13 people with single-sided deafness tried to participate in the survey. Data from the German CI registry report that 19% of CI surgeries in 2022 were performed on patients who had normal hearing in the contralateral ear (Stöver et al., 2024). This shows a great interest of this relatively small (but growing) patient group in the topic and the need for instruments that evaluate quality of life in this group. CI for single-sided deafness has an impact on people's quality of life (Oh et al., 2023) but it might be different than for people with bilateral hearing loss. The development of the CIQOL instruments did not include adults with single-sided deafness. Further research should investigate whether the instruments are able to detect important changes in hearing-related quality of life in this group. Otherwise, it might be necessary to adapt the instruments or develop new instruments for people with single-sided deafness.

### Study Strengths and Limitations

The validation of a new PROM is crucial to ensure that it consistently measures the intended construct. This study used a variety of statistical measures to present a comprehensive picture of the reliability and validity of the German language version of the CIQOL instruments. This ensures researchers and clinicians who aim to measure CI users’ hearing-related quality of life that they can rely on these instruments.

Limitations of this study mainly concern the study sample and recruitment methods. In any study, there is a risk of self-selection bias where people who are interested in research about the topic or who have strong opinions may be those most likely to participate in research studies. Overall, we were able to recruit a sample of adults with hearing loss with various demographic and hearing loss characteristics. The demographics of the CI population in German speaking countries have not been thoroughly studied and remain largely unknown. Evaluation of demographic data from the German CI registry of about 2,100 patients implanted in 2022 gives first insights into the CI population in Germany (Stöver et al., 2024). Most surgeries were conducted with patients over 18 years. Of those, about 40% were aged between 65 and 85 years, about 39% were aged between 45 and 65 years and 3% were aged over 85 years. About 50% of the patients were male and 50% female (Stöver et al., 2024). With 26% of CI users aged between 65 and 85, 44% aged between 45 and 65 years, and only 0.5% above 85, the sample in the present study was younger, and with 65% more female CI users were represented. German was the first language for 89% of the adult CI patients in Germany (Stöver et al., 2024), in our sample it was even more with 98%. Overall, we find similar patterns in the sample that was registered in the CI registry in 2022 and the participants of the present study. There is a tendency that more women, younger people and people with German as their first language participated in this survey. However, the comparability of demographic data is limited because we included any people who use CI and data of the CI registry only included people who had CI surgery in 2022. This may explain for example, why the percentage of people with postlingual onset of hearing loss was as high as 90% of CI surgeries in 2022 (Stöver et al., 2024) whereas in the present study only 77% of CI users stated their hearing loss was acquired.

There are some limitations to conducting an online survey and recruiting the sample through the distribution of flyers via CI centers, rehabilitation clinics, and CI associations. Using an online survey excludes people without access to online devices. However, in Germany, 92% of private households own at least one computer, 94% of households have an internet connection and 91% of people over the age of 10 use the internet (as of 2019) (Statistisches Bundesamt [Destatis] et al., 2021). The study purposely included people who have bilateral hearing loss who do not (yet) use CI. As the questionnaire will be used for people before their CI surgery, it should be applicable in this group, too. In our sample, only very few participants stated not to use CI. This might be a result of the distribution of information on study participation mainly through CI clinics and associations. Although people with indication for CI may be affiliated with these institutions, they might be preoccupied with the decision about a CI, or they might not feel addressed by a study with a CI quality of life questionnaire. The small sample of people without a CI limited our abilities to perform comparisons between groups of participants regarding their hearing devices. The possibility of recruiting a large sample of adults with bilateral hearing loss outweighed the limitations of the recruitment methods. However, future studies could try to explicitly address CI candidates who do not yet have a CI to recruit more people from this group. This would enable a comparison between groups.

The fixed order of the questionnaires in the online survey with first the demographics, second the CIQOL-35 Profile, and third the NCIQ may have resulted in higher missing response rates in the last questionnaire. However, we decided to place the questionnaire known to be the longest at the end of the survey, to avoid losing participant's interest in the beginning of the survey. After consultation with our ethics committee, we included a statement before the NCIQ section of the survey, stating that some items or terms used in the NCIQ may be no longer adequate. We explained that it would still be very important for our study that they respond to all items. This could have influenced participants’ responses. We hope that the comment helped to reduce participant burden and made participants more willing to respond to as many items as possible.

Another methodical limitation is that we did not assess test-retest reliability as an additional reliability measure. We focused on calculating internal consistency, because it minimizes the effort for participants and recruitment methods and is not affected by potential external influences (e.g., memory effects, mood fluctuations, or contextual changes between administrations). In the original validation study, the CIQOL showed very strong test-retest reliability (McRackan et al., 2021).

It was beyond the scope of the study to examine the sensitivity of the instruments to various clinical variables, such as speech recognition scores, duration of CI use, before and after cochlear implantation, or wearing time. Therefore, future studies with the German CIQOL instruments should examine the sensitivity to important clinical variables.

## Conclusions

Based on our findings, the adapted German version of the CIQOL instruments is reliable and valid, confirming it as a valuable measure for quality of life in adults with CI that even has some advantages over the NCIQ. Clinicians and researchers can use the German CIQOL instruments to gain more comprehensive information on everyday experiences from CI users. This enables clinicians to provide intervention and support on most needed topics. Ongoing research on CI users’ quality of life will help to improve interventions with CI and consultation of CI candidates. Availability of the instruments in various languages provides the opportunity to share, compare and unite results from research studies from different regions in the world and thereby widen the evidence base for CI practice.

## Supplemental Material

sj-docx-1-tia-10.1177_23312165261450792 - Supplemental material for Assessing Hearing-Related Quality of Life in Adults With Hearing Loss: Validation of the German Cochlear Implant Quality of Life (CIQOL)-35 Profile Supplemental material, sj-docx-1-tia-10.1177_23312165261450792 for Assessing Hearing-Related Quality of Life in Adults With Hearing Loss: Validation of the German Cochlear Implant Quality of Life (CIQOL)-35 Profile by Elena Pützer, Theodore R. McRackan, Isabelle Boisvert, Judy R. Dubno and Karolin Schäfer in Trends in Hearing

## Abbreviations

CFA: Confirmatory Factor Analysis
CFI: Comparative Fit Index
CI: Cochlear Implant
CIQOL: Cochlear Implant Quality of Life
COSMIN: COnsensus-based Standards for the selection of health Measurement INstruments
DGHNO-KHC: Deutsche Gesellschaft für Hals-Nasen-Ohren-Heilkunde, Kopf- und Hals-Chirurgie e.V
DWLS: Diagonal weighted least squares
ERSA: Évaluation du Retentissement de la Surdité chez l’Adulte
hAVICOP: Hearing-related quality of life questionnaire for auditory-visual, cognitive and psychosocial functioning
HL-QOL: Quality of Life in People with Hearing Loss Questionnaire
HUI-3: Health Utility Index
ICF: International Classification of Functioning, Disability, and Health
LEQ-CI21: Listening Effort Questionnaire-Cochlear Implant
MuRQoL: Music-Related Quality of Life Measure
NCIQ: Nijmegen Cochlear Implant Questionnaire
PROM: Patient-Reported Outcome Measure
RMSEA: Root Mean Square Error of Approximation
SFL: Standardized Factor Loading
SRMR: Standardized Root Mean Square Residual
TLI: Tucker-Lewis Index
WHO: World Health Organization
