# Supplementary material for: Assessing Hearing-Related Quality of Life in Adults With Hearing Loss: Validation of the German Cochlear Implant Quality of Life (CIQOL)-35 Profile
Source: Trends Hear. 2026 Jun 16;30:23312165261450792. doi: 10.1177/23312165261450792 (PMC13272987; doi:10.1177/23312165261450792)
Supplement: sj-docx-1-tia-10.1177_23312165261450792 - Supplemental material for Assessing Hearing-Related Quality of Life in Adults With Hearing Loss: Validation of the German Cochlear Implant Quality of Life (CIQOL)-35 Profile [file sj-docx-1-tia-10.1177_23312165261450792.docx]

| **Supplement 1** | | |  |  |  |
| --- | --- | --- | --- | --- | --- |
| *Comparison of convergent validity with non-parametric and parametric analyses and with the validation of the English version* | | | | | |
| CIQOL Domain | NCIQ Domain / Subdomain | Spearman Rho (*r_s_*) | Pearson (r) | English validation Pearson (r) | Difference between English and German |
| Communication | NCIQ Total | **.752** | **.774** | **.81** | .036 |
|  | Physical | **.728** | **.766** | **.780** | .014 |
|  | Basic sound perception | .598 | .673 | **.700** | .027 |
|  | Advanced sound perception | **.757** | **.779** | **.830** | .051 |
|  | Speech production | .552 | .553 | .420 | -.133 |
| Emotional | NCIQ Total | **.740** | **.756** | **.730** | -.026 |
|  | Psychological | **.796** | **.802** | **.800** | -.002 |
|  | Self-esteem | **.796** | **.802** | **.800** | -.002 |
| Entertainment | NCIQ Total | .665 | .674 | .680 | .006 |
| Environment | NCIQ Total | **.724** | **.744** | **.730** | -.014 |
|  | Basic sound perception | **.750** | **.782** | n/a | n/a |
| Listening Effort | NCIQ Total | **.725** | **.740** | **.730** | -.010 |
| Social | NCIQ Total | **.757** | **.770** | **.770** | .000 |
|  | Social | **.740** | **.759** | **.720** | -.039 |
|  | Activity limitation | .683 | .698 | **.700** | .002 |
|  | Social interaction | **.742** | **.767** | .650 | -.117 |
| CIQOL-10 Global | NCIQ Total | **.848** | **.846** | **.850** | .004 |
| CIQOL-10 Global | NCIQ Short version | **.825** | **.821** | n/a | n/a |
| Mean (min.- max.) | | | | | .028 (.000-.133) |
| *Note.* Strong correlations are highlighted in **bold**. | | |  |  |  |
